# Understanding HAT1: A Comprehensive Review of Noncanonical Roles and Connection with Disease

**DOI:** 10.3390/genes14040915

**Published:** 2023-04-14

**Authors:** Miguel A. Ortega, Diego De Leon-Oliva, Cielo Garcia-Montero, Oscar Fraile-Martinez, Diego Liviu Boaru, María del Val Toledo Lobo, Ignacio García-Tuñón, Mar Royuela, Natalio García-Honduvilla, Julia Bujan, Luis G. Guijarro, Melchor Alvarez-Mon, Miguel Ángel Alvarez-Mon

**Affiliations:** 1 Department of Medicine and Medical Specialities, Faculty of Medicine and Health Sciences, University of Alcalá, 28801 Alcala de Henares, Spain; diego.leon@edu.uah.es (D.D.L.-O.);; 2 Ramón y Cajal Institute of Sanitary Research (IRYCIS), 28034 Madrid, Spain; 3 Cancer Registry and Pathology Department, Principe de Asturias University Hospital, 28806 Alcala de Henares, Spain; 4 Department of Biomedicine and Biotechnology, University of Alcalá, 28801 Alcala de Henares, Spain; 5 Unit of Biochemistry and Molecular Biology, Department of System Biology (CIBEREHD), University of Alcalá, 28801 Alcala de Henares, Spain; 6 Immune System Diseases-Rheumatology, Oncology Service and Internal Medicine (CIBEREHD), University Hospital Príncipe de Asturias, 28806 Alcala de Henares, Spain

**Keywords:** HAT1, acetyltransferase, histones H3H4, epigenetics, noncanonical roles, cancer, viral infections, immunoinflammatory diseases, translational opportunities

## Abstract

Histone acetylation plays a vital role in organizing chromatin, regulating gene expression and controlling the cell cycle. The first histone acetyltransferase to be identified was histone acetyltransferase 1 (HAT1), but it remains one of the least understood acetyltransferases. HAT1 catalyzes the acetylation of newly synthesized H4 and, to a lesser extent, H2A in the cytoplasm. However, 20 min after assembly, histones lose acetylation marks. Moreover, new noncanonical functions have been described for HAT1, revealing its complexity and complicating the understanding of its functions. Recently discovered roles include facilitating the translocation of the H3H4 dimer into the nucleus, increasing the stability of the DNA replication fork, replication-coupled chromatin assembly, coordination of histone production, DNA damage repair, telomeric silencing, epigenetic regulation of nuclear lamina-associated heterochromatin, regulation of the NF-κB response, succinyl transferase activity and mitochondrial protein acetylation. In addition, the functions and expression levels of HAT1 have been linked to many diseases, such as many types of cancer, viral infections (hepatitis B virus, human immunodeficiency virus and viperin synthesis) and inflammatory diseases (chronic obstructive pulmonary disease, atherosclerosis and ischemic stroke). The collective data reveal that HAT1 is a promising therapeutic target, and novel therapeutic approaches, such as RNA interference and the use of aptamers, bisubstrate inhibitors and small-molecule inhibitors, are being evaluated at the preclinical level.

## 1. Introduction

Chromatin consists of DNA and histones. Histones are proteins that are critical to the assembly and compaction of the genetic material in the nucleus. Histones are organized in octamers formed by tetramers of H3-H4 and two H2A-H2B dimers, and DNA wraps the octamer (147 bp in 1.75 turns) to form a nucleosome [1,2,3,4,5]. Post-translational modifications (PTMs) of proteins regulate their functions and, thus, various biological processes [6]. Hundreds of distinct PTMs have been characterized, and among them, the most thoroughly studied is phosphorylation. However, histone and nonhistone protein acetylation is another extensively studied PTM regulated by histone acetyltransferases (HATs) and histone deacetylases (HDACs) [7]. Moreover, the acetylation of proteins regulates many biological processes, such as apoptosis, cytoskeletal dynamics, DNA damage repair, chromatin structure, transcriptional regulation and cellular metabolism, and it has been linked with pathologies such as cancer and neurological disorders [8,9,10]. Acetylation is a mark that activates the expression of the genes wrapped around a histone because the acetyl group neutralizes the positive charge of the histone and reduces the affinity of its binding to DNA.

Histone acetyltransferase 1 (HAT1) is a type B HAT that belongs to the Gcn5-related N-acetyltransferase (GNAT) family. The first function ascribed to HAT1 was catalysis of the rapid transfer of an acetyl group from acetyl coenzyme A exclusively onto the ε-amino group of lysine (K) 5 and K12 in the amino-terminus of an H4 histone immediately after it was synthesized in the cytosol and before the assembly of chromatin [11]. This modification pattern has been largely conserved among all eukaryotes, in contrast with the low percentage of H3 proteins that undergo acetylation after synthesis, which is not a conserved process in eukaryotes [12]. Moreover, the function of this mark is not well understood because it is removed 20 min after H3 is incorporated into the chromatin [12,13]. HAT1 is encoded by *HAT1*, located on 2q31.1, and carries 12 exons. It is highly conserved across a variety of eukaryotes. HAT1 is the only known instance of a type B histone acetyltransferase that is essential for acetylating freshly produced histones. Although less frequently, it also acetylates H2A at K5 [14].

In addition to its first role, described in the cytoplasm, HAT1 is principally located in the nucleus, and it acetylates nonhistone proteins, such as mitochondrial proteins, chromatin-related proteins and transcription factors.

The first acetyltransferase discovered was HAT1; however, it is still one of the GNAT family members that is least understood. In this review, we discuss the enzymatic function of HAT1 and its noncanonical functions to understand the broad role played by HAT1 in cellular processes. The associations between HAT1 expression levels and functions are then reviewed. Finally, we describe recent therapeutic approaches to target HAT1.

## 2. Biochemical Features of HAT1

**Figure 1:**
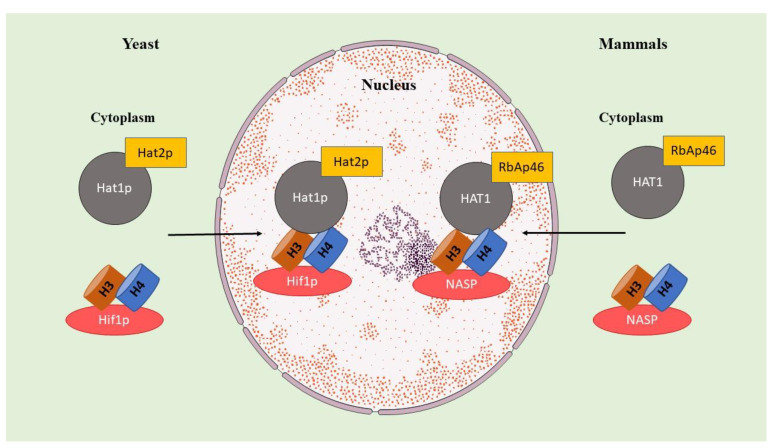
Scheme of complexes of HAT1 in nucleus (NuB4 complex) and cytoplasm compared in mammals and yeast. HAT1: histone acetyltransferase 1, NASP: nuclear autoantigenic sperm protein, RbAp46: Retinoblastoma protein-associated protein 46.

HAT1 forms two complexes. In the cytoplasm of mammalian cells, HAT1, or its homolog in yeast, Hat1p, forms a complex with RbAp46 (or its homolog Hat2p in yeast). The nuclear complex with HAT1, named NuB4, also includes a histone chaperone, nuclear autoantigenic sperm protein (NASP) in mammals or Hif1p in yeast (see Figure 1) [15].

Three domains constitute HAT1’s elongated shape: an N-terminal domain with residues 23–136, a central domain with residues 137–270 and a C-terminal domain with residues 271–341. The canyon formed between the central and C-terminal domains is bound by the HAT1 cofactor acetyl coenzyme A (Ac-CoA). The H4 peptide adopts a well-known structure with two *β*-turns at sites including glycine (G), leucine (L) and lysine residues in the HAT1-Ac-CoA-H4 ternary complex: G7-K8-G9-L10 and L10-G11-K12-G13 [16].

HAT1 is expressed as two transcriptional isoforms, named HAT1*a* and HAT1*b* in normal human keratinocytes, formed by 418 and 334 amino acids, respectively [17]. The specific roles played by these isomers remain to be elucidated. Interestingly, the majority of both isomers localize to the nucleus, with HAT1*b* localizing to a lesser extent to the cytoplasm.

Initially, no effects were observed in yeast lacking HAT1. This finding indicated that the cell expresses a functionally redundant protein. This supposition was supported when HAT1 knocked down in combination with the introduction of specific N-terminal H3 mutations led to reduced DNA damage sensitivity and attenuated telomeric silencing [18,19,20].

The absence of HAT1 in knockout murine models led to neonatal lethality because of impaired lung development due to hyperproliferation of mesenchymal cells, leading to severe atelectasis, reduced aeration and death via respiratory failure [11]. This finding suggested faulty cell fate decisions. However, *Drosophila melanogaster* HAT1 mutants exerted only a slight effect on viability; did not affect analyzed adult phenotypes, such as those related to daily activity, sleep pattern or heat-stress tolerance; and exerted no effect on fertility [21].

In vivo studies of HAT1 led to the identification of several phenotypes associated with the loss of its enzymatic activity. These traits, which include defective heterochromatin gene silencing, reduced DNA damage sensitivity, genome instability and alterations in gene expression, are likely due to the histone-modifying function of HAT1 [22].

## 3. Noncanonical Functions of HAT1

**Figure 2:**
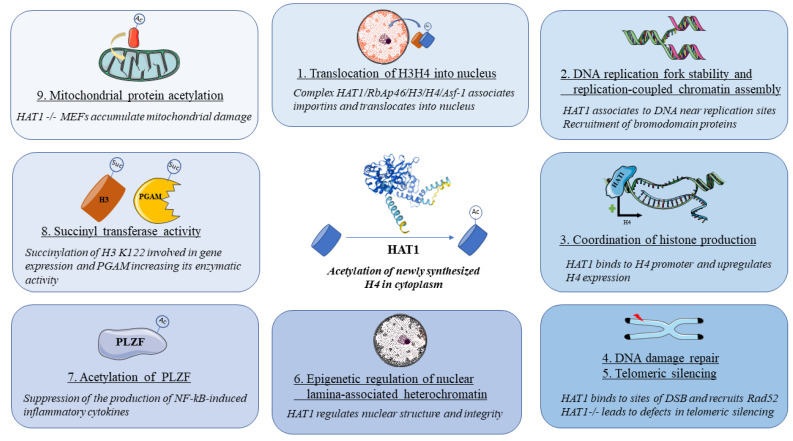
Overview of canonical and noncanonical functions of HAT1. Center: canonical role of HAT1 consists of the acetylation of newly synthesized H4 histones in the cytoplasm. Peripheral: non-canonical roles of HAT1 encompass: (1) Translocation of H3H4 into nucleus, (2) DNA replication fork stability and replication-coupled chromatin assembly, (3) Coordination of histone production, (4) DNA damage repair, (5) Telomeric silencing, (6) Epigenetic regulation of nuclear lamina-associated heterochomatin, (7) Acetylation of PLZF, (8) Succinyl transferase activity and (9) Mitochondrial protein acetylation. HAT1 = histone acetyltransferase 1, RbAp46 = pRB-associated protein p46, Asf-1 = anti-silencing factor 1, DSB = double-strand break, PLZF = promyelocytic leukemia zinc finger, PGAM = phosphoglycerate mutase, MEF = mouse embryonic fibroblast.

HAT1 fulfills both canonical and noncanonical functions. The former is represented by the enzymatic acetylation of K5 and K12 in the N-terminus of H4 in a newly assembled H3H4 dimer in the cytoplasm [23]. Nevertheless, the fact that most HAT1 molecules are found in the nucleus led to the supposition that this enzyme is involved in functions in addition to the acetylation of the newly synthesized histone H4 called noncanonical functions and are summarized below (see Figure 2).

### 3.1. Translocation of Newly Synthesized Histone H3H4 into the Nucleus

HAT1 forms a complex with the histone chaperone RbAp46 (Hat2p in yeast) in the cytoplasm. RbAp46 binds helix 1 in the H4 C-terminus. HAT1 acetylates K5 and K12 in the H4 N-terminus. The complex then associates with the histone chaperone Asf1, to which it transfers H3H4. The Asf1/H3/H4 complex associates with the importins and karyopherins that translocate into the nucleus, where H3 is then acetylated, and the dimers are assembled by Caf1 through its physical interaction with PCNA [24,25].

Surprisingly, HAT1 is apparently more stable in complex with its substrate after acetylation [26]. Moreover, di-acetylation may mediate histone import into the nucleus. However, the experiments by Agudelo et al. with mouse embryonic fibroblasts (MEFs) suggested that HAT1 is not required for histone import and deposition into chromatin because they did not detect differences in the amount of histones in the cytoplasm and nucleus in *HAT1−/−* MEFs [27]. We propose two scenarios. The first is that the role of HAT1 in histone translocation and deposition may be complementary or redundant with the other proteins involved, and thus HAT1 is not essential for this process. The second is that the absence of HAT1 activates alternative mechanisms where other histone acetylases are recruited to fulfill the role of HAT1.

### 3.2. DNA Replication Fork Stability and Replication-Coupled Chromatin Assembly

During replication, parental histones must be removed from the chromatin and reassembled with the newly synthesized histones after the replication fork has duplicated the DNA. The replisome dissociates the histone octamer into an H3H4 tetramer and two H2AH2B dimers [28]. Agudelo et al. showed that HAT1 physically and transiently associates with DNA near the replication sites and that the loss of HAT1 inhibits the progression of the replication fork, increases the incidence of replication fork stalling, destabilizes stalled forks and induces the MRE11-dependent degradation of newly synthesized DNA [29]. Hence, HAT1 may play a role in the stabilization of newly replicated DNA and chromatin assembly.

On the one hand, according to Barman et al., for replication-coupled chromatin assembly, HAT1 is not necessary because these authors did not observe defects in cell viability in HAT1-deficient DT40 cells [30]. On the other hand, according to data from Agudelo et al., HAT1-dependent acetylation of K5 and K12 in H4 appears to be involved in the recruitment of a subset of bromodomain proteins, including Brg1 (an ATP-dependent chromatin-remodeling enzyme), Baz1A and Brd3 [27]. Motifs called bromodomains identify and attach to acetylated lysine residues. The Brg1 level is reduced by 50%, and the Baz1A and Brd3 levels are reduced by approximately 25–30% in the chromatin of *HAT1 −/−* MEFs [27]. Additionally, Agudelo et al. showed that HAT1 is necessary for nascent chromatin to acquire the correct topology. They observed an increase in the levels of topoisomerase 2α and topoisomerase 2β in the chromatin in the absence of HAT1. These authors hypothesized that this increase is due to the incorrect formation of the nucleosomes, leading to the recruitment of topoisomerase 2, which can resolve the topological defects in the chromatin structure. Finally, they observed that the dissociation of HAT1 from the chromatin preceded the deacetylation of H4 K5 and K12; thus, HAT1 may be involved in the regulation of chromatin maturation by maintaining the H4 acetylation mark.

### 3.3. Coordination of Histone Production

According to the evidence reported by Gruber et al., HAT1 is required for the EGF-dependent proliferation of telomerase-immortalized human mammary epithelial cells (hTert-HME1) but not in the absence of EGF. As the scientists looked into the biochemical process by which HAT1 promotes cell growth, they discovered that HAT1 attaches to the histone H4 gene’s promoter and triggers the production of new H4 histones. In addition, it was shown that HAT1 expression is essential for cell cycle progression into the S phase. Gruber et al. also demonstrated that the H4 promoter is sensitive to the level of acetate because of a “H4 box” motif, rendering H4 sensitive to glucose level, which is metabolized to acetyl-CoA in the proliferating cells [31]. Thus, they described a nutrient-sensing role for HAT1.

### 3.4. DNA Damage Repair

An initial genetic analysis of yeast showed that the loss of HAT1 expression leads to no effects on cell viability or chromatin assembly [32]. However, the loss of HAT1 in combination with mutations in specific sets of lysine residues in the histone H3 NH2-terminus results in defective telomeric silencing and sensitivity to methyl methanesulfonate, which damages DNA [19,20]. Indeed, *HAT1 −/−* MEFs exhibit multiple defects, including slow growth, DNA damage sensitivity and genome instability (untreated *HAT1 −/−* cells show numerous γ-H2AX foci) [11], indicating that HAT1 may play a role in DNA damage repair.

Employing an inducible HO endonuclease system followed by chromatin immunoprecipitation (ChIP), the factor recruitment to double-strand breaks (DSBs) can be monitored. Experiments showed that Hat1p and HIF1 (a homolog of the mammalian protein NASP) are recruited near DSBs with kinetics similar to those of the recombinational repair factor Rad52 in yeast [33]. Moreover, Yang et al. found that HAT1 facilitates the recruitment of the repair factor Rad51 by incorporating H4K5/K12-acetylated H3.3 (H3.3-H4K5/12ac) into DSB sites mediated through its HIRA-dependent histone turnover activity and promotes homologous recombination-mediated DNA repair [34]. Finally, the nuclear HAT1 complex (NuB4), composed of Hat1p/Hat2p (RbAp46 in mammals), Hif1p (an H3H4-specific histone chaperone) and histones H3 and H4, has been shown to participate in DNA repair-linked chromatin reassembly [35].

### 3.5. Telomeric Silencing

Telomeric silencing is the outcome of the transcriptional repression of genes near heterochromatic telomeric structures [36]. Experiments with *Saccharomyces cerevisiae*, Kelly et al. demonstrated that the loss of Hat1p combined with substitution mutations in which lysine residues are replaced with arginine residues in the N-terminus of the H3 histone (which maintains a basic charge and prevents acetylation of the N-terminus) leads to defective telomeric silencing [19]. The fact that the combination of these two alterations is necessary indicates that HAT1 and H3 histones show redundant functions that are required for telomeric silencing. Moreover, the experiments by Mersfelder et al. contributed details explaining HAT1 function in telomeric silencing by suggesting that HAT1 must be catalytically active and located in the nucleus [37].

### 3.6. Epigenetic Regulation of Nuclear Lamina-Associated Heterochromatin

An assay for transposase-accessible chromatin using sequencing (ATAC-seq) with MEFs revealed that HAT1 loss results in a reduction in genome accessibility at 1895 sites. These sites, called HAT1-dependent accessibility domains (HADs), are located mostly in the distal intergenic regions with low GC content and low gene enrichment, and they range in size from 0.9 kb to 11 Mb and present characteristics of heterochromatin [38]. Subsequent experiments demonstrated that HADs localize within lamina-associated domains (LADs; with 86% overlapping). Moreover, HADs show an 85% overlap with constitutive LADs; hence, large domains associated with the nuclear lamina’s accessibility are regulated by HAT1. Explaining the mechanisms underlying this HAT1 regulatory function, HAT1 is shown to be a global repressor of H3 K9 methylation and that the HADs correspond to regions where HAT1 regulates the abundance of H3 K9 methylation. The loss of HAT1 results in increases in nuclear size, similar to the effect of decreased lamina expression or mutations that compromise lamina function [38]. These findings show that HAT1 is a regulator of nuclear structure and integrity.

### 3.7. NF-κB Response Moderation by Regulating the Transcription Factor PLZF

Sadler et al. described a novel role for HAT1, namely, the acetylation of the transcriptional regulator promyelocytic leukemia zinc finger protein (PLZF), a critical cofactor of the NF-kB transcriptional complex that inhibits damaging inflammation [39]. PLZF binds to specific motifs in gene promoters and recruits transcription cofactors, thereby functioning either as an inducer or repressor, depending on the covalent modification [40]. HAT1 activation during innate immune signaling was discovered by Sadler et al. to be mediated by calcium/calmodulin-dependent protein kinase (CaMK2), showing that it acetylates PLZF at K227. PLZF then forms a repressive complex by binding with HDAC3 and the NF-κB p50 subunit on the promoter of NF-kB-regulated genes and leads to the suppressed production of NF-kB-induced inflammatory cytokines [39].

### 3.8. Succinyl Transferase

Yang et al. described a novel role for HAT1 in HepG2 cell lines, in which HAT1 functions as the lysine-succinyl transferase of histone and nonhistone proteins. In HAT1-depleted cells, the succinylation level of 324 sites in 204 proteins is inhibited, and these proteins are widely localized and participate in multiple cellular pathways. Therefore, HAT1-mediated succinylation may affect global cellular processes. Among the proteins thus modified, the H3 succinylated at K122 (involved in gene expression and epigenetic regulation) and phosphoglyceromutase 1 (PGAM1) succinylated at K99 stand out. PGAM1 K99 succinylation shows increased enzymatic activity and is associated with enhanced glycolysis during tumorigenesis [41]. HAT1 involvement in tumorigenesis is further reviewed below.

### 3.9. Mitochondrial Protein Acetylation

Recent investigations have focused on the link between HAT1 and the acetylation of mitochondrial proteins. The acetylation of mitochondrial proteins is involved in regulated mitochondrial dynamics, namely, fusion and fission [42], and metabolic enzymes, including those involved in fatty acid oxidation and ketone body production [43]. However, whether these processes are catalyzed by enzymes or result from the metabolism of acetyl-CoA in the mitochondria remains unclear [44,45].

By performing subcellular fractionation and mitochondrial purification, Agudelo-Garcia et al. demonstrated that HAT1 exhibits mitochondrial localization, despite HAT1 lacking a mitochondrial localization signal [22]. In addition, according to Marin et al., adenosine monophosphate-activated protein kinase (AMPK) phosphorylates HAT1, DNA methyltransferase 1 (DNMT1) and RbAp46 in human umbilical vein endothelial cells (HUVECs). This triggers the inhibition of DNMT1 and the activation of HAT1 and leads to important nucleosomal remodeling. This results in the upregulation of the nuclear genes that encode the proteins involved in mitochondrial biogenesis and function, including transcription factor A (Tfam), uncoupling proteins 2 and 3, peroxisome proliferator-activated receptor γ coactivator-1a (PGC-1a) and (UCP2 and UCP3) [46]. However, the results from Nagarajan et al. indicated that HAT1 does not affect the expression of the aforementioned transcription factors; therefore, the effects must be cell specific. Notably, HAT1*−/−* MEFs show accumulated mitochondrial damage, and this research group proposed that HAT1 must therefore play a more direct role in mitochondrial regulation [47]. They also created a haploinsufficient mouse model (HAT1 +/−) with a shortened lifespan and an early-onset aging phenotype that comprised lordokyphosis, muscular atrophy, minor growth retardation, decreased subcutaneous fat, cancer and paralysis. Notably, mitochondrial dysfunction is a hallmark of aging [48,49].

## 4. Clinical Relevance

Because of the central role played by HAT1 in several physiological processes, HAT1 dysregulation is involved in a broad spectrum of pathologies, such as cancer, viral infections and inflammatory diseases [50,51,52,53]. In this section, the main implications of HAT-1 action in these maladies are presented.

### 4.1. HAT1 and Cancer

Cancer constitutes a group of diseases in which cells undergo uncontrolled and self-sufficient proliferation. Hanahan and Weinberg identified six hallmarks of cancer in 2000: cancer cells show self-sufficient proliferation mediated by growth signaling, insensitivity to anti-growth signaling, tissue invasion and metastasis, limitless replicative potential and apoptosis evasion, and in tumors, angiogenesis is sustained [54]. In 2011, the same authors added four more hallmarks: deregulation of cellular energetics, avoiding immune destruction, genome instability and tumor-promoting inflammation [55]. Finally, in 2022, Hanahan added four new hallmarks: unlocked phenotypic plasticity, nonmutational epigenetic reprogramming, polymorphic microbiome establishment and cell senescence [56]. These hallmarks were described as driving forces in tumorigenesis. In this context, HAT1 participates in DNA damage repair, telomeric silencing and other hallmarks of cancer [19,27,34]. Many studies have evaluated the expression of HAT1 across different types of tumors. In the majority of these tumors, the high expression of HAT1 is associated with poor outcomes, as assessed by Gruber et al. in the available TCGA data identifying six types of solid tumors: adrenocortical carcinoma (ACC), bladder urothelial carcinoma (BLCA), kidney chromophobe (KICH), brain lower grade glioma (LGG), liver hepatocellular carcinoma (LIHC) and lung adenocarcinoma (LUAD) [31] (see Table 1). However, in some cancers, such as lung cancer and melanoma, the downregulation of HAT1 contributes to apoptosis and therapy resistance [50,57]. For all of these reasons, HAT1 is an interesting histone modifier to investigate for a better understanding of cancer, as is discussed below.

#### 4.1.1. HAT1 in the Evasion of the Immune Response

Studies have identified a potential role of HAT1 in the evasion of immune responses that are mediated via well-established mechanisms. For instance, Fas (also known as CD95) is a cell death receptor that initiates apoptosis when it binds to the Fas ligand in cytotoxic immune cells [58]. Studies with lung cancer cells have demonstrated that the HAT1 and Fas expression levels are lower than those in normal lung cells, and these expression levels are positively correlated [50]. Specifically, the overexpression of protease-activated receptor 2 (PAR2) inhibits the expression of HAT1 and Fas. The restoration of HAT1 expression restores Fas expression and induces lung cancer cell apoptosis. These results suggest that HAT1 is an interesting target for lung cancer treatment.

On the other hand, the studies from Fan et al. showed that HAT1 is overexpressed and associated with poor prognosis in pancreatic ductal adenocarcinoma [59]. Indeed, they demonstrated that in pancreatic cancer cell lines, HAT1 is overexpressed and a growth-promoting factor. Furthermore, the expression of HAT1 is correlated with the expression of programmed death-ligand 1 (PD-L1), a ligand for the programmed death 1 (PD1) receptor, which induces the apoptosis of T cells and an underlying mechanism of the immune resistance of the tumor cells [60,61]. Moreover, subsequent experiments revealed that HAT1 upregulates PD-L1 expression through the action of the transcription factor BRD4, which binds to the promoter of PD-L1.

Finally, HAT1 plays a regulatory role in CD4+Foxp3+ regulatory T cells (Tregs), a special type of lymphocyte involved in maintaining the balance of immunoinflammatory responses [62]. Previous works have revealed that a specific population of infiltrating Tregs in breast cancer expresses the chemokine receptor CCR4. Tumor cells and tumor-associated macrophages secrete the chemokines CCL22 and CCL17, which attract CCR4+ Treg cells [63]. On the basis of these underlying mechanisms, the group showed that FOXP3 bound to the *CCR4* promoter recruits HAT1, which acetylates the CCR4 promoter, leading to CCR4 receptor upregulation and Treg infiltration. Collectively, these studies support an immunomodulatory role for HAT1 in cancer, making HAT1 an important player to study in carcinogenesis.

#### 4.1.2. HAT1 in Cancer Chemoresistance

HAT1 has also been related to chemoresistance in different types of tumors. For example, gemcitabine is the treatment of choice for nonresectable PDAC tumors. However, gemcitabine resistance develops within weeks of treatment [64]. To explain the chemoresistance in PDAC, a methyltransferase, an enhancer of Zeste homolog-2 (EZH2), a catalytic subunit of Polycomb repressive complex 2 (PRC2) and the long noncoding RNA (lncRNA) PVT1 have been identified [65]. EZH2 maintains cancer stem cells and regulates the expression of PVT1, an inducer of chemoresistance. Sun et al. showed that HAT1 binds to the N-terminus of EZH2, increasing the stability of EZH2 because HAT1 prevents the interaction of EZH2 with the E3 ubiquitin ligase UBR4. Moreover, HAT1 increases the expression of PVT1 by facilitating BRD4 binding to the *PVT1* promoter [51]. Furthermore, the nanoparticles carrying siHAT1 restore gemcitabine sensitivity in vitro and in vivo.

Other lines of research have revealed a relationship between HAT-1 and hepatocellular carcinoma (HCC) treatment with cisplatin, which is commonly associated with drug resistance in patients with this cancer [66]. Notably, during the development of cisplatin resistance, many changes at the genetic and epigenetic levels are evident [67]. HAT1 has been shown to contribute to cisplatin resistance. For example, HAT1 is found to be elevated in HCC cells. Furthermore, HAT1 promotes the Warburg effect, in part by changing the expression of the enzymes involved in glucose metabolism [68]. Knocking down HAT1 via transfection with short hairpin RNAs (shRNAs) sensitizes HCC cells to apoptotic death induced by cisplatin. It seems that the multiple roles of HAT1 in combination with increased aerobic glycolysis contribute to the development of cisplatin resistance in HCC.

On the other hand, Bugide et al. recently demonstrated that the BRAFV600E melanoma cells treated with a BRAF inhibitor (BRAFi) downregulate the expression of HAT1, and by employing NanoString-based nCounter PanCancer Pathway Panel-based gene expression analysis, they found that the MAPK, Ras, transforming growth factor (TGF)-β and Wnt pathways are activated [57]. The MAPK pathway is significantly increased via insulin growth factor 1 receptor (IGF1R) signaling, and treatment with an ERK inhibitor and IGF1R inhibitor restores the BRAFi sensitivity in cells lacking HAT1 [57]. However, how HAT1 knockdown activates these pathways was not explained. Further studies with melanoma cells showed that cells pretreated with ascorbate (vitamin C) show increased sensitivity to BET inhibitors (BETis). Ascorbate downregulates the expression of HAT1, probably promoting DNA demethylation in the HAT1 gene region. The decrease in H4 acetylation reduces the binding sites of the BRD proteins [69]. Thus, it has been proposed that the ascorbate levels in melanoma patients be taken into account to reduce the dosage of BETis and thereby attenuate BETi side effects.

In addition, HAT1 is highly expressed in prostate cancer cells, and its overexpression is related to castration-resistant prostate cancer (CRPC) progression and the expression of androgen receptors (ARs), including full-length AR and AR variant 7 [70]. AR variants mediate the resistance to enzalutamide (ENZ) treatment [71]. In this case, the participation of BRD4 in the oncogenic processes that mediate AR expression via the recognition of H4 acetylation marks has been shown. Knocking down the expression of HAT1 with ascorbate restores cell sensitivity to ENZ [70].

#### 4.1.3. HAT1 in Carcinogenesis Based on Tumor Type

HAT1 is also involved in other critical events related to cancer development and progression. In studies of nasopharyngeal cancer (NPC), Miao et al. found that HAT1 is overexpressed, and regulates the expression of the antiapoptotic protein Bcl2-like protein 12 (Bcl2L12), inducing resistance to apoptosis [72]. Yang et al. concluded that HAT1 succinyltransferase activity is essential for tumorigenesis [41]. HAT1 promotes tumorigenesis by the succinylation of H3K122, modulating epigenetic regulation and PGAM, contributing to enhanced glycolysis.

**Table 1:** HAT1 expression levels, divided by upregulation or downregulation, and functions in different types of cancer.

Types of Cancer	Expression Level	Target	Mechanism of Action			References
DLBCL, ACC, BLCA, KICH, LGG, LIHC and LUAD	Upregulated		Expression of HAT1		Low survival outcomes	[31,73]
PDAC	Upregulated	PD-L1	Evasion of immune response		BRD4 binds to H4 acetylated by HAT1 and initiates transcription of PD-L1	[59]
Breast cancer	Upregulated	CCR4			FOXP3/HAT1 binds the *CCR4* promoter and upregulates *CCR4* expression, leading to Treg infiltration	[63]
Pancreatic cancer	Upregulated	lncRNA PVT1	Chemoresistance	Gemcitabine	HAT1 enhances PVT1 expression by facilitating BRD4 binding to PVT1 promoter	[51]
		EZH2			HAT1 increases stability of EZH2, preventing the latter from interacting with the E3 ubiquitin ligase UBR4	[65]
HCC	Upregulated	-		Cisplatin	Knocking down HAT1 sensitizes HCC cells to cisplatin-induced apoptosis	[68]
Melanoma	Upregulated	H4ac		BETi	Ascorbate reduces HAT1 expression, leading to a decrease in H4K5 and H4K12 acetylation, and impairs the binding of BRD4 to acetylated histones, restoring BETi sensitivity	[69]
CRPC	Upregulated	AR		ENZ	Upregulates AR expression via a BRD4-mediated pathway	[71]
NPC	Upregulated	Bcl2L12	Other roles		HAT1 upregulates Bcl2L12, leading to the apoptosis resistance of NPC cells	[72]
HepG2 cell line	Upregulated	H3K122			H3K122 succinylation by HAT1 activates oncogene expression	[41]
		PGAM			PGAM succinylation enhanced glycolysis	
Lung cancer	Downregulated	Fas	Evasion of immune response		Downregulation of HAT1 leads to downregulation of Fas	[50]
Melanoma	Downregulated	IGF1R signaling	Chemoresistance	BRAFi	Loss of HAT1 drives BRAFi resistance by activating MAPK signaling mediated via IGF1R	[57]

### 4.2. HAT1 and Viral Infections

In addition to the abovementioned information about the involvement of HAT1 in chromatin assembly and epigenetic regulation, recent studies have linked the expression and function of HAT1 with the hepatitis B virus (HBV), human immunodeficiency virus infections and antiviral protein viperin synthesis (see Table 2).

#### 4.2.1. HBV Infection

The roles of HAT1 in the replication and epigenetic regulation of the HBV covalently closed circular DNA (cccDNA) minichromosome have been explored. By performing RNA interference (RNAi)-based screening, Wang et al. demonstrated that HAT1 regulates HBV replication, showing that the loss of HAT1 or KAT8 (a lysine acetyltransferase) reduces HBV-DNA and pregenomic RNA levels in HepG2.2.15 cells [74]. HAT1 and KAT8 may control HBV replication by means of the transcription factors hepatocyte nuclear factor 4 (HNF4) and peroxisome proliferator-activated receptor γ coactivator-1 (PPARGC-1), according to the lowered levels of these HBV replication regulators [74].

In addition, Yang et al. established a human liver chimeric mouse model and identified some key roles of HAT1 in the regulation of cccDNA minichromosome formation [52]. Initially, the HBV-infected cells show a significantly elevated expression of HAT1, CAF-1 and the lncRNA HULC; therefore, the group concluded that HAT1 contributes to HBV replication and cccDNA accumulation. Additionally, HAT1 expression is upregulated by the HBV X protein (HBx)-controlled transcription factor Sp1. Finally, HAT1/CAF-1 was found to contribute to the assembly of the HBV cccDNA minichromosome mediated via the acetylation of H4K5 and H4K12. HAT1 is recruited to the cccDNA minichromosomes in the HCC context by the action of the upregulated lncRNA HULC-scaffold hepatitis B core antigen (HBc), promoting histone acetylation in this process [52].

#### 4.2.2. HIV Infection

The human immunodeficiency virus (HIV) integrates into the host DNA and generates a dual-effect epigenetic environment; that is, both the host and viral genomes influence the expression of each other [75]. Hence, a study of enzyme expression and global DNA methylation in individuals chronically infected with HIV-1 revealed that HAT1 expression is upregulated on CD4+ T cells [76]. Additionally, another study associated monocyte immune dysfunction from untreated and treated HIV+ patients with epigenetic changes. As a monocyte- and macrophage-specific scavenger receptor (CD163) that is shed into plasma, soluble CD163 (sCD163) is a recently discovered plasmatic HIV progression biomarker linked with poor outcomes [77]. A study identified that HAT1 is the epigenetic biomarker most highly correlated with HIV patients expressing high sCD163 levels [78]. Therefore, it seems that HAT1 plays an important epigenetic role in HIV infection and may be a potential therapeutic target.

#### 4.2.3. Host Antiviral Defense

Type I interferon (IFN) production is stimulated during the innate immune response against viral infections [79]. A protein called viperin (virus inhibitory protein, endoplasmic reticulum associated, interferon inducible) has targeted the antiviral action that can be activated both by IFN and without it [80]. HAT1 induces acetylation of the K197 of viperin, leading to the recruitment of UBE4A, which in turn stimulates K6-linked polyubiquitination at K206 of viperin. Finally, polyubiquitination results in viperin protein degradation, which the authors suggested may involve proteasomes. Moreover, HAT1 knockdown inhibits UBE4A-induced viperin ubiquitination, and HAT1 overexpression downregulates the viperin protein levels [81].

### 4.3. HAT1 and Immunoinflammatory and Vascular Diseases

HAT1 has been linked to inflammation through the modulation of the NF-κB response by regulating the action of transcription factor PLZF (see 3.8, NF-κB response moderation by regulating the transcription factor PLZF). Therefore, HAT1 plays an important role in immunoinflammatory diseases such as chronic obstructive pulmonary disease (COPD), atherosclerosis and the vascular disease related to ischemic stroke, in which the immune response also plays an important role in pathogeneses and outcomes (see Table 2).

#### 4.3.1. HAT1 Regulates the TLR4 Signaling Pathway in COPD

Recent evidence points to microRNAs (miRNAs) as the key regulators in the immunopathogenesis of COPD [82,83,84,85]. Zhang et al. reported that miR-486-5p is upregulated in alveolar macrophages and peripheral monocytes, in which it targets the expression of *HAT1* mRNA and leads to an increase in the Toll-like receptor 4 (TLR4) level. Moreover, the expression of miR-486-5p is positively correlated with the expression levels of the inflammatory cytokines IL-6, IL-8, TNF-α and IFN-γ [86]. Hence, HAT1 may downregulate the expression of TLR4 and diminish the inflammatory response in COPD.

#### 4.3.2. HAT1 Promotes Reactive Oxygen Species (ROS) Production and Cholesterol Accumulation in Macrophages in Atherosclerosis

A dual role for HAT1 has been described in the development of atherosclerosis. On the one hand, HAT1 contributes to ROS overproduction in macrophages [87]. In atherosclerotic samples, Nox5, p300 (a type A HAT), HAT1 and H3K27ac (an epigenetic marker of active gene expression) are upregulated and located within macrophage-rich areas. Moreover, cultured human macrophages stimulated with lipopolysaccharide (LPS) exhibit upregulated HAT1 and Nox5 expression, which leads to histone acetylation mediated via the activation of TLR4-dependent signaling pathways and the recruitment of p300 and HAT1 to transcription-activating sites within the proximal promoter of the *Nox5* gene [87]. Taken together, these studies indicated that inflammation-promoting signaling triggered by TLR4 may activate HATs in macrophages, inducing chromatin structure remodeling and leading to increased expression of Nox5 and subsequent ROS production.

On the other hand, HAT1 participates in the transformation of macrophages into foam cells via miR-486, because in is involved in the regulation of cholesterol efflux in macrophages and downregulation of ATP-binding cassette transporter A1 (ABCA1), a transporter of cholesterol [53]. Altogether, miR-486 seems to accelerate the transformation of foam cells by targeting HAT1.

#### 4.3.3. HAT1 Participates in Regulating the Protein Expression Profile after Ischemic Stroke

After photothrombotic stroke (PTS) in the rat brain cortex, an array of changes in the expression of several proteins may be regulated by epigenetic mechanisms [88,89]. In penumbra tissue, the expression of either the histone deacetylases HDAC1, HDAC2 and HDAC4 or that of the histone acetyltransferases HAT1 and p300/CBP-associated factor (PCAF) is upregulated, while the expression of H3K9ac is downregulated [90]. Therefore, the synthesis of proteins may be suppressed and activated by HDACs and HATs, respectively. Further research showed that HAT1 and PCAF are mostly found in the nuclei of astrocytes and neurons, respectively, and that the augmentation of their expression after PTS is linked to a rise in the cytoplasm of the neurons and, particularly, the astrocytes, which do not colocalize with apoptotic cells [91]. Thus, HAT1 may be involved in the regulation of the response to the injured brain either in the neurons or the astrocytes.

**Table 2:** HAT1 expression and function in viral infections and immunoinflammatory and vascular diseases.

Pathology		Expression	Target	Mechanism of Action	References
Viral infections	HBV	Upregulated	HNF4α and PPARGC-1-α	HAT1 regulates HBV replication via these transcription factors	[74]
			H4K5 and H4K12	HAT1 is recruited by HULC-HBc to cccDNA and acetylates H4K5 and H4K12, what induces the assembly of the HBV cccDNA minichromosome	[52]
	HIV	Upregulated	sCD163	HAT1 is the best epigenetic biomarker of HIV-infected monocytes	[77]
Immunoinflammatory/vascular diseases	COPD	Downregulated	TLR4	miR-486-5p targets HAT1, and TLR4 is upregulated and triggers an inflammatory response	[86]
	Atherosclerosis	Upregulated	Nox5	TLR4 signaling activates HAT1, leading to upregulation of Nox5, which leads to ROS production	[87]
		Downregulated	ABCA1	miR-486 downregulates HAT1, leading to downregulation of ABCA1, increasing the accumulation of lipids in macrophages, which are transformed into foam cells	[53]
	Ischemic stroke	Upregulated		Altered synthesis of proteins in penumbra tissue	[90,91]

## 5. Translational Opportunities

The role of HAT1 in tumorigenesis is not yet well understood. However, due to the available data, HAT1 has become an attractive target for the rational design of inhibitors that block its activity and induce cell cycle arrest or apoptosis in tumor cells.

Xue et al. performed RNAi screening to identify the genes involved in regulating the proliferation of Eca-109 human esophageal carcinoma (EC) cells by using a lentiviral shRNA library. They determined that knocking down HAT1 leads to G2/M cell cycle arrest and inhibits proliferation [92]. The underlying mechanism seems to be the inhibition of ERK and Akt phosphorylation, which leads to the downregulation of cyclin D1 and decreases the level of cyclin B1, thus regulating the progression from G2 to mitosis [93]. They also demonstrated that the expression of HAT1 is higher in primary tumor cells than in normal esophageal tissue cells [92].

Small single-stranded DNA or RNA oligonucleotides called aptamers are synthesized in vitro by a procedure called the systematic evolution of ligands by exponential enrichment (SELEX). Aptamers acquire a tertiary conformation and bind to their targets on the basis of shape complementarity at the aptamer–target interface with high affinity and specificity, modulating the function of their targets (proteins, whole cells or organs); therefore, they are promising new diagnostic and therapeutic tools [94]. Klett-Mingo et al. obtained an apHAT610 aptamer after six rounds of screening and showed that it inhibits the acetyltransferase activity of HAT1 and exhibits antitumoral properties in lung cancer cell lines. In particular, it prevents colony formation, triggers apoptosis and stops the cell cycle [95].

Bisubstrate inhibitors are compounds comprising two conjugated fragments, each of which is targeted to a site in an enzyme that catalyzes a reaction, mimicking the ternary complex of an aptamer. These inhibitors are reported to show an increased affinity for a targeted molecule because more interactions are possible [96]. Ngo et al. synthesized a set of peptide-CoA conjugates using the N-(9-fluorenyl) methoxycarbonyl (Fmoc)-based solid-phase peptide synthesis protocol. The bisubstrate inhibitor H4K12CoA is composed of the first 20 amino acids of H4 and coenzyme A added to K12 and shows a low Ki value of 1.1 nM [97]. Inhibition at this submicromolar concentration may lead to the development of new drugs that target HAT1 in important diseases such as those described herein. However, H4K12CoA does permeate cells, which limits its utility.

In addition, some authors have proposed a high-throughput, peptide-based, click chemistry-enabled enzymatic assay for the design of new inhibitors. However, the results are unpublished [98]. Overall, the main translational opportunities are summarized in Table 3.

## 6. Conclusions

The first role ascribed to HAT1, the acetylation of newly synthesized H4, has been described as a cytoplasmic process. However, HAT1 has been shown to localize predominantly in the nucleus. Moreover, a broad spectrum of HAT1 regulatory activities have recently been discovered in key cellular processes. Indeed, the expression and function of HAT1 have been linked with diseases that affect the global population, such as cancer, viral infections and inflammatory diseases (COPD, atherosclerosis and ischemic stroke). Assessing these links may lead to the use of HAT1 as a novel biomarker and therapeutic target. Further research to better understand the multiple pathways in which HAT1 is involved will lead to more accurate therapeutic approaches.

## Figures and Tables

**Table 3:** Main translational opportunities targeting HAT1.

Inhibitor Molecules of HAT1	Pathology	Mechanism of Action	Synthesis Method	References
RNAi	EC	Knocks down HAT1	RNAi screening using a lentiviral short hairpin RNA (shRNA) library	[92]
Aptamers	Lung cancer cell lines	Inhibits acetyltransferase activity	SELEX	[95]
Bisubstrate inhibitors		Inhibits acetyltransferase activity	Fmoc-based solid-phase peptide synthesis protocol	[97]
Small-molecule inhibitors		Inhibits acetyltransferase activity	Acetyl-click screening platform	[98]

## Data Availability

Not applicable.
